# Late‐Onset Spondyloarthritis Presenting as Glucocorticoid‐Resistant Polymyalgia Rheumatica: A Hitherto Underappreciated Entity in Which Tumor Necrosis Factor or Interleukin ‐17 Blockade May Have a Therapeutic Role

**DOI:** 10.1002/art.43320

**Published:** 2025-12-08

**Authors:** Kerem Abacar, Gabriele De Marco, Jake Weddell, Katya Meridor, Tom Macleod, Andrew Scarsbrook, Kulveer Mankia, Edward Vital, Andrew Barr, Colin Pease, Helena Marzo‐Ortega, Sarah L. Mackie, Dennis McGonagle

**Affiliations:** ^1^ Leeds Institute of Rheumatic and Musculoskeletal Medicine, University of Leeds, Chapel Allerton Hospital Leeds United Kingdom; ^2^ NIHR Leeds Biomedical Research Centre, Leeds Teaching Hospitals NHS Trust Leeds United Kingdom; ^3^ Rheumatology Department, Tel Aviv Sourasky Medical Center, Affiliated with Tel Aviv University Faculty of Medicine Tel Aviv Israel; ^4^ Leeds Institute of Health Research, St James's University Hospital, University of Leeds Leeds United Kingdom

## Abstract

**Objective:**

Polymyalgia rheumatica (PMR) is an age‐related inflammatory disease with shoulder/hip girdle involvement. Magnetic resonance imaging (MRI) reveals extracapsular/entheseal soft tissue involvement in both PMR and spondyloarthritis (SpA), with sacroiliac joint and perientheseal spinal bone marrow edema (BME) being characteristic of SpA. Therefore, some shared anatomic topography might be expected to result in similar clinical features. Herein, we describe the clinical and imaging features of SpA initially diagnosed as PMR.

**Methods:**

Patients observed at Leeds Teaching Hospitals NHS Trust with a diagnosis of psoriatic arthritis (PsA) or axial SpA were screened to identify those initially diagnosed with PMR from 2002 to 2024. Only those patients who retrospectively fulfilled the 2012 EULAR/American College of Rheumatology classification criteria or the Bird et al criteria for PMR were included. Clinical data relevant to initial PMR diagnosis, imaging features, follow‐up, and treatment data were collected, as well as radiographic or MRI features that established the final diagnosis.

**Results:**

Thirty‐one patients (median age 62 [interquartile range (IQR) 58–69] years; 17 women and 14 men) presenting with typical PMR shoulder/hip girdle pain were subsequently classified as having SpA spectrum disorders. The SpA diagnosis was made in 12 patients within three months of presentation and in 19 patients during the remaining follow‐up period (median 3 [IQR 1–4] years). Four of 27 tested patients were HLA–B27 positive. BME on MRI was detected in the spine and/or sacroiliac joints in 20 of 25 patients (80%) who underwent imaging (sacroiliac joint: 17 patients [68%]; spine: 15 patients [60%]). Clinical resolution with C‐reactive protein (CRP) level normalization occurred in 21 of 31 patients following initial glucocorticoid (GC) therapy, but 7 of these 21 initial responders experienced disease flares or elevations in CRP levels. Therapy‐wise, disease‐modifying antirheumatic drugs (DMARDs) were used in 21 of 31 patients: 8 received conventional synthetic DMARDs, and 11 received biologic agents (8 anti–tumor necrosis factor agents, 3 interleukin‐17 inhibitors), whereas the remaining 10 patients were treated with ≤10 mg/day of GCs.

**Conclusion:**

Late‐onset SpA with PMR clinical presentations is characterized by failure to respond to or taper GC therapy and is often identified by SpA‐specific osteitis patterns on MRI. We propose that a PMR–SpA overlap may account for biologic therapy efficacy in steroid‐refractory PMR.

## INTRODUCTION

Polymyalgia rheumatica (PMR) is a chronic, age‐related inflammatory rheumatic disease characterized by symmetric pain and stiffness in the hip and shoulder girdles and sometimes the neck alongside elevated levels of acute‐phase reactants.^1^ The diagnosis is clinical. PMR typically responds rapidly and completely to medium doses of glucocorticoids (GCs) (15–20 mg of prednisolone) but many relapse during subsequent taper, which may prompt diagnostic re‐evaluation.^2^ “PMR mimics” include nonrheumatic diseases (eg, malignancy) and other inflammatory rheumatic diseases such as rheumatoid arthritis (RA), spondyloarthritis (SpA), and giant cell arteritis (GCA).^2^ This diagnostic ambiguity has long been recognized, and previous frameworks have outlined the clinical assessment of patients with polymyalgic symptoms to ensure mimics are carefully considered.^3^ The 2012 PMR classification criteria were developed using RA as the most common comparator, and absence of peripheral synovitis or a negative rheumatoid factor or anti–cyclic citrullinated peptide antibody result became part of these criteria.^4^ In clinical practice, the acceptance of PMR as a clinical diagnosis, the extent of disability from untreated PMR, the rapidity of response to GCs, and the opportunity to re‐evaluate the diagnosis at a later date limit the role of imaging in the initial diagnostic workup.

Extracapsular involvement detected by magnetic resonance imaging (MRI) or fluorine‐18 fluorodeoxyglucose–positron emission tomography/computed tomography (FDG‐PET/CT) scanning can assist in the differentiation of PMR from other conditions.^5^, ^6^ Unlike PMR, SpA tends to present in younger adults and has specific imaging characteristics frequently used in the diagnosis. In a study evaluating the utility of FDG‐PET/CT to differentiate PMR and SpA, FDG uptake in the shoulder, ischial tuberosity, and interspinous process regions was seen in both conditions, although somewhat more often in PMR.^7^ Axial SpA (axSpA) pathology is characterized by a similar tissue topography on MRI and PET/CT, with extracapsular changes similar to PMR,^8^, ^9^, ^10^ but SpA additionally shows florid bone marrow edema (BME) and osteitis, which are typical of SpA.^11^ Hence, anatomic overlap and anatomic distinctions are observed across PMR and SpA.

As illustrated by many case reports, the intertwined manifestations of both diseases under the definition of late‐onset SpA presenting with polymyalgic symptoms can challenge the diagnostic expertise of clinicians.^12^ The recent findings indicate that late‐onset axSpA represents a distinct phenotype with lower rates of HLA–B27 positivity, family history of SpA, and inflammatory back pain, while showing higher rates of peripheral arthritis. Furthermore, in both late‐onset and young‐onset ankylosing spondylitis groups, anti–tumor necrosis factor (anti‐TNF) therapy yields comparable improvements in disease activity and functional status, with initial response rates in the late‐onset group catching up to those in the young‐onset group by 12 and 24 months.^13^, ^14^

Of crucial importance is the fact that SpA involvement is linked to osteitis, whereas PMR is generally not, and this offers a useful contemporary imaging cue to differentiate between SpA and PMR.^15^ Furthermore, some phenotypic clues might trigger clinicians to consider SpA in the differential of PMR, most notably the absence of a vigorous initial and sustained PMR response to low‐dose GC therapy. In this study, we aimed to characterize patients within the late‐onset SpA spectrum who initially presented with PMR‐like symptoms using clinical and imaging features; we identified two distinct groups: one in which SpA diagnosis was made promptly and another in which the diagnosis was significantly delayed. Our findings call for the consideration of an uncommon but difficult PMR clinical presentation that actually represents late‐onset SpA.

## METHODS

### Patients

To systematically identify patients who initially presented with PMR‐like symptoms and were later diagnosed with SpA, we performed a structured search of electronic clinical correspondence at the Leeds Teaching Hospitals NHS Trust. The search strategy incorporated both PMR‐related terms (eg, “PMR,” “polymyalgia,” “shoulder stiffness,” “girdle pain”) and SpA‐associated terms (eg, “spondyloarthritis,” “spondylitis,” “psoriasis,” “enthesitis,” “uveitis,” “inflammatory bowel disease (IBD),” “HLA‐B27”, “sacroiliitis”). This approach enabled the retrospective identification of cases in which features of both disease spectra had been documented at any point, irrespective of the initial diagnostic label. Patients who retrospectively fulfilled 2012 EULAR/American College of Rheumatology (ACR) polymyalgia rheumatica classification criteria^4^ or the Bird et al criteria for PMR^16^ at the time of initial presentation were included. Among them, individuals who were also diagnosed—either at baseline or during follow‐up with axSpA (including both radiographic axSpA [r‐axSpA] and nonradiographic axSpA), axial psoriatic arthritis (PsA), or enteropathic arthritis with axial involvement were selected for final analysis. The Assessment of SpondyloArthritis international Society (ASAS) classification criteria^17^ were used for axSpA classification, except for the age criterion due to the late‐onset nature of this cohort. PsA diagnosis was based on the Classification of Psoriatic Arthritis (CASPAR) criteria,^18^ and patients classified as having enteropathic arthritis had a confirmed diagnosis of IBD established by a gastroenterologist.

Some of the patients initially presented with PMR and also exhibited certain features suggesting axSpA. These patients, whose axSpA diagnosis was mainly driven by extra‐articular symptoms within the first three months, were categorized as “diagnosed with SpA within the first three months.” Patients who received a diagnosis of SpA beyond the initial three‐month period—typically following clinical reassessment prompted by persistent inflammatory back pain or failure to taper GCs—were categorized as “diagnosed with SpA at the follow‐up time.” Baseline demographic data of the patients (such as age, sex, body mass index, familial history, etc), clinical features, imaging and laboratory data at presentation and during follow‐up, and treatments received were recorded retrospectively from the patients’ charts.

### Imaging data

#### Conventional radiography

Conventional radiographs were performed on all patients with imaging of the sacroiliac joint (SIJ) or spine or both at the time of suspicion of an alternative SpA diagnosis because SIJ imaging is not usually undertaken for suspected PMR. The classification of patients as having r‐axSpA was based on the 1984 modified New York criteria by a rheumatology expert (KA).^19^

#### MRI and other imaging

All MRI findings regarding axial and peripheral joint involvement at or after the diagnosis of PMR were recorded. SIJ and spinal magnetic resonance images of the patients performed when SpA diagnosis was suspected were evaluated. MRI was performed either immediately after the initial diagnosis of PMR (to exclude SpA in the differential diagnosis) or in cases in which GC treatment could not be stopped or tapered or in the presence of flare‐ups suggestive of SpA during GC treatment. Sacroiliitis classification on MRI was assessed according to ASAS criteria.^20^ FDG‐PET/CT was performed during the diagnostic process of PMR, especially to exclude large vessel vasculitis or neoplasia, but on occasion showed evidence suggestive of SpA, which was evaluated after the clinical SpA diagnosis emerged.

### Statistical analyses

Data were analyzed using Statistical Package for the Social Sciences version 22.0 (SPSS). Continuous variables were expressed as mean (SD) and median (interquartile range [IQR]) for normal and nonnormal distribution, respectively. Frequency (percentage) was used for the description of categorical variables.

This retrospective study focused on the overlapping PMR and SpA populations and was conducted as an approved retrospective service evaluation (audit) at the specialist rheumatology clinic of Leeds Teaching Hospitals Trust (the formal registration number of this audit is LOC1428). Data are available on reasonable request.

## RESULTS

Thirty‐one patients (median age 62 [IQR 58–69] years, female:male ratio 17:14) presenting with PMR symptoms and subsequently SpA spectrum disorders were diagnosed (the annual diagnosis numbers of the patients are exhibited in the Supplementary Figure). All patients were >50 years of age at diagnosis and presented with typical PMR symptoms (symmetric shoulder and hip girdle pain plus intense stiffness). The frequency of constitutional symptoms in our cohort (29%) was lower than expected (typically reported as 94.8% in PMR).^21^ All patients fulfilled the 2012 ACR/EULAR PMR classification criteria,^4^ except three with normal C‐reactive protein (CRP) levels at baseline. However, these patients were included because imaging clearly showed the same pathology pattern in PMR with normal acute‐phase reactant response.^2^ Of 13 patients who had baseline FDG‐PET/CT scanning for potential concomitant vasculitis, 8 had features of PMR on imaging, including shoulder/pelvic girdle and interspinous FDG uptake (Table 1). One patient exhibited features of GCA, and only two patients showed FDG uptake in the SIJs. Patients were observed for a median of 6 (IQR 3.8–10.3) years, and in this period, concurrent GCA occurred in five (16.1%) of them.

**Table 1:** Comparison of clinical and imaging features of patients diagnosed with SpA in the first three months and patients diagnosed with SpA in the follow‐up period*

	All patients (N = 31)	Diagnosed with SpA in the first 3 mo (n = 12)	Diagnosed with SpA at follow‐up (n = 19)
Age at PMR presentation, median (IQR), y	62 (58–69)	64 (57–67)	62 (59–70)
Sex (female/male)	17/14	6/6	11/8
Shoulder and hip girdle pain/stiffness, n (%)	31 (100)	12 (100)	19 (100)
Neck pain, n (%)	23 (74.2)	7 (58.3)	16 (84.2)
Constitutional symptoms, n (%)	9 (29)	2 (16.7)	7 (36.8)
Psoriasis, n (%)	7 (22.6)	5 (41.7)	2 (10.5)
Peripheral arthritis, n (%)	5 (16.1)	4 (33.3)	1 (5.3)
Familial history of SpA, n (%)	5 (16.1)	5 (41.7)	0 (0)
Inflammatory bowel disease, n (%)	4 (12.9)	2 (16.7)	2 (10.5)
Uveitis, n (%)	2 (6.5)	2 (16.7)	0 (0)
Peripheral enthesitis, n (%)	5 (16.1)	3 (25)	2 (13.3)
HLA–B27 positivity,^a^ n (%)	4 (14.8)	1 (9.1)	3 (18.8)
Diabetes mellitus, n (%)	7 (22.6)	2 (16.7)	5 (26.3)
CRP at presentation, median (IQR), mg/L	43 (23–54)	37.5 (23.3–69)	43 (16.2–53)
CRP at third month of first GC treatment, median (IQR), mg/L	5 (5–10)	5 (5–16.7)	5.6 (5–9.7)
Normal CRP at third month of first GC treatment, n (%)	16 (51.6)	7 (58.3)	9 (47.4)
Bone marrow edema in SIJ on MRI, n (%)	17 (68)	6 (66.7)	11 (68.8)
Structural changes in SIJ on MRI, n (%)	15 (60)	4 (44.4)	11 (68.8)
Bone marrow edema in spine on MRI, n (%)	15 (60)	4 (50)	11 (64.7)
Radiographic sacroiliitis, n (%)	14 (46.7)	4 (33.3)	10 (55.6)
PMR findings on PET/CT scan, n (%)	8 (61.5)	1 (50)^b^	7 (63.6)
Complete GC response in first 3 mo, n (%)	21 (67.7)	7 (58.3)	14 (73.7)
Relapse during GC tapering, n (%)	14 (45.2)	4 (33.3)	10 (52.6)
Received csDMARDs, n (%)	16 (51.6)	5 (41.7)	11 (57.9)
Received bDMARDs, n (%)	14 (45.2)	6 (50)	8 (42.1)
Concomitant giant cell arteritis, n (%)	5 (16.1)	1 (8.3)	4 (21.1)
Normal CRP at the last visit, n (%)	22 (71)	10 (83.3)	12 (63.2)
Receiving GC at the last visit, n (%)	11 (35.5)	3 (25)	8 (42.1)

* bDMARD, biologic disease‐modifying antirheumatic drug; CRP, C‐reactive protein; csDMARD, conventional synthetic DMARD; GC, glucocorticoid; IQR, interquartile range; MRI, magnetic resonance imaging; PET/CT, positron emission tomography/computed tomography; PMR, polymyalgia rheumatica; SIJ, sacroiliac joint; SpA, spondyloarthritis.

^a^ Data were available for 27 patients.

^b^ Only two patients had PET/CT imaging.

### Subgroups (diagnosed with SpA in the first three months and diagnosed with SpA at the follow‐up time)

Among the 31 patients with PMR presentation, 12 (38.7%) were diagnosed with SpA within the first three months of their initial outpatient visit (Table 1). Factors associated with early SpA recognition were the presence of psoriasis and a family history of SpA. The remaining 19 (61.3%) patients were diagnosed with SpA after a median duration of 3 years (IQR 1–4 years) because protracted musculoskeletal symptoms and the inability to taper GCs triggered imaging investigations (Table 1). Although the sample size was insufficient for statistical analysis, findings that are used for ASAS SpA classification and that also facilitate the clinical diagnosis of SpA, such as psoriasis (41.7% vs 10.5%), a positive family history of SpA (41.7% vs 0%), and uveitis (16.7% vs 0%), were detected more frequently in the “diagnosed with SpA in the first three months” (n = 12) group than in the “diagnosed with SpA at the follow‐up time” (n = 19) group. Additionally, SpA‐related findings were more frequently observed on imaging in patients in the “diagnosed with SpA at the follow‐up time” group (Table 1). Furthermore, the use of biologic disease‐modifying antirheumatic drugs (bDMARDs) was more prevalent among patients “diagnosed with SpA in the first three months,” whereas the use of GCs and conventional synthetic DMARDs (csDMARDs) was less common at the time of the last visit (Table 1).

### SpA features

The median delay in SpA diagnosis was 3 (IQR 1–4) years in the “diagnosed with SpA at the follow‐up time” group. Seven patients could be classified as having PsA, five of whom had psoriasis diagnoses before PMR diagnosis, and four patients could be classified as having IBD, two of whom had IBD diagnoses before PMR diagnosis. Sacroiliac MRI revealed SIJ BME in 17 patients and structural changes (fatty infiltration, erosion, sclerosis, ankylosis) in 15 patients. Among the 25 patients examined with spinal MRI, BME was detected in the spine and SIJs in 20 (80%) patients and spinal BME was detected in 15 (60%) patients. Among these, SIJ BME was present in 16 patients diagnosed during follow‐up and in 9 patients diagnosed within the first three months; spinal BME was detected in 17 and 8 patients, respectively; and structural abnormalities in the spine were noted in 17 and 7 patients, respectively. Additionally, MRI showed soft tissue entheseal/capsular changes only (overlapping with PMR on imaging) in five patients, making SpA diagnosis reliant on other features given that interspinous and other entheseal/capsular tissues are the target for both SpA and PMR. However, syndesmophyte formation attributable to SpA was seen in only one patient. Additionally, 14 of 31 patients were classified as having r‐axSpA based on conventional radiographic imaging, whereas the remaining 17 patients were classified as having nonradiographic axSpA.

### Treatments

**Figure 1:**
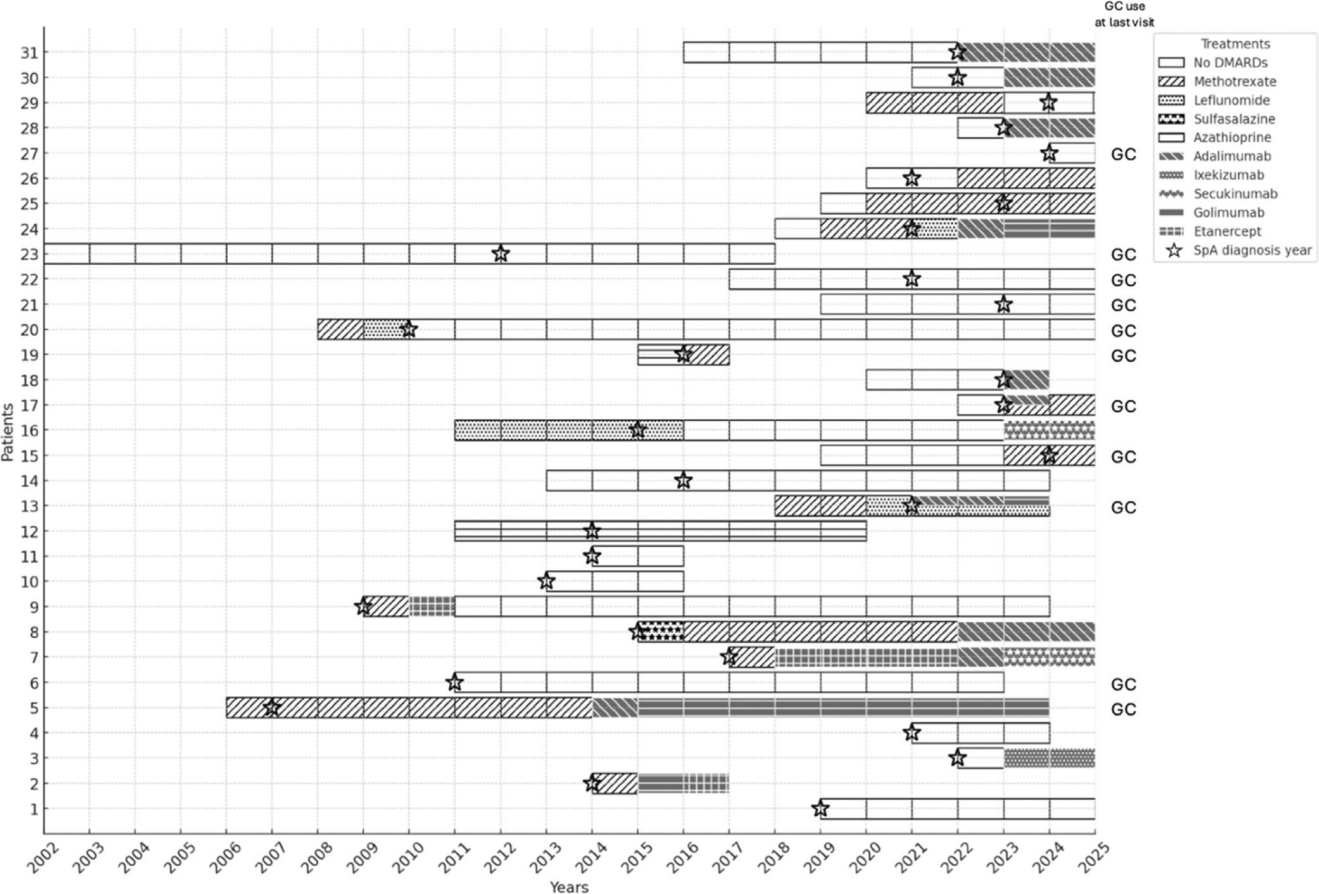
Treatmentregimens of the patients. The changes in immunosuppressive treatments received by all patients (n = 31) over time are shown. Initially, all patients were started on GC treatment, and csDMARD and bDMARD treatment changes were made during the follow‐up. Six of these patients started csDMARDs and bDMARDs at the time of diagnosis of SpA. At the last examination, 10 patients were continuing their GC treatment at a dose below 10 mg/day. bDMARD, biologic disease‐modifying antirheumatic drug; csDMARD, conventional synthetic DMARD; GC, glucocorticoid; SpA, spondyloarthritis.

At the time of initial diagnosis, all 31 patients with PMR at presentation were started on GC therapy at dosages of 15 to 20 mg/day. In the 5 of 31 cases in which PMR was accompanied by GCA, a higher GC dosage of 20 to 60 mg/day was administered. The PMR symptoms of 21 (67.7%) patients completely resolved after the first GC treatment, whereas 10 patients showed a clinically partial response to GC treatment. Among the patients with high CRP levels at baseline (n = 28), CRP levels improved by the first three months after GC treatment in 16 cases. Fourteen of 21 patients who achieved complete remission with initial GC therapy had no relapse during follow‐up (median 5 [IQR 2.8–10.3] years). The remaining seven patients were started on DMARD treatment during follow‐up due to relapse. Two patients were directly started on a bDMARD, whereas the other five patients were first treated with csDMARDs, but two of them were started on bDMARDs after relapse with csDMARDs (Figure 1).

Of the 10 patients who achieved partial clinical remission with initial GC treatment, one was directly started on bDMARD treatment, and the other nine were started on csDMARD treatment. Seven of nine patients initially started on csDMARDs were later switched to bDMARDs. However, bDMARD treatment was stopped after 60 months of treatment due to remission in only one case. Patients who had a relapse during their first csDMARD treatment and were started on a second csDMARD (n = 7) were switched to bDMARD treatment after experiencing a relapse (Figure 1). At the last visit, 12 of 31 patients were not receiving DMARD treatment. Seven patients were receiving methotrexate (MTX), one was receiving azathioprine, three were receiving golimumab, five were receiving adalimumab, two were receiving secukinumab, and one was receiving ixekizumab (Figure 1). Eleven patients were still receiving GC therapy, and in 22 patients, the most recent CRP level was within normal limits. Only 6 of 31 patients had stopped all therapy and were discharged. Additionally, among the five patients who developed GCA, three were treated with MTX and one received anti‐TNF therapy.

## DISCUSSION

**Figure 2:**
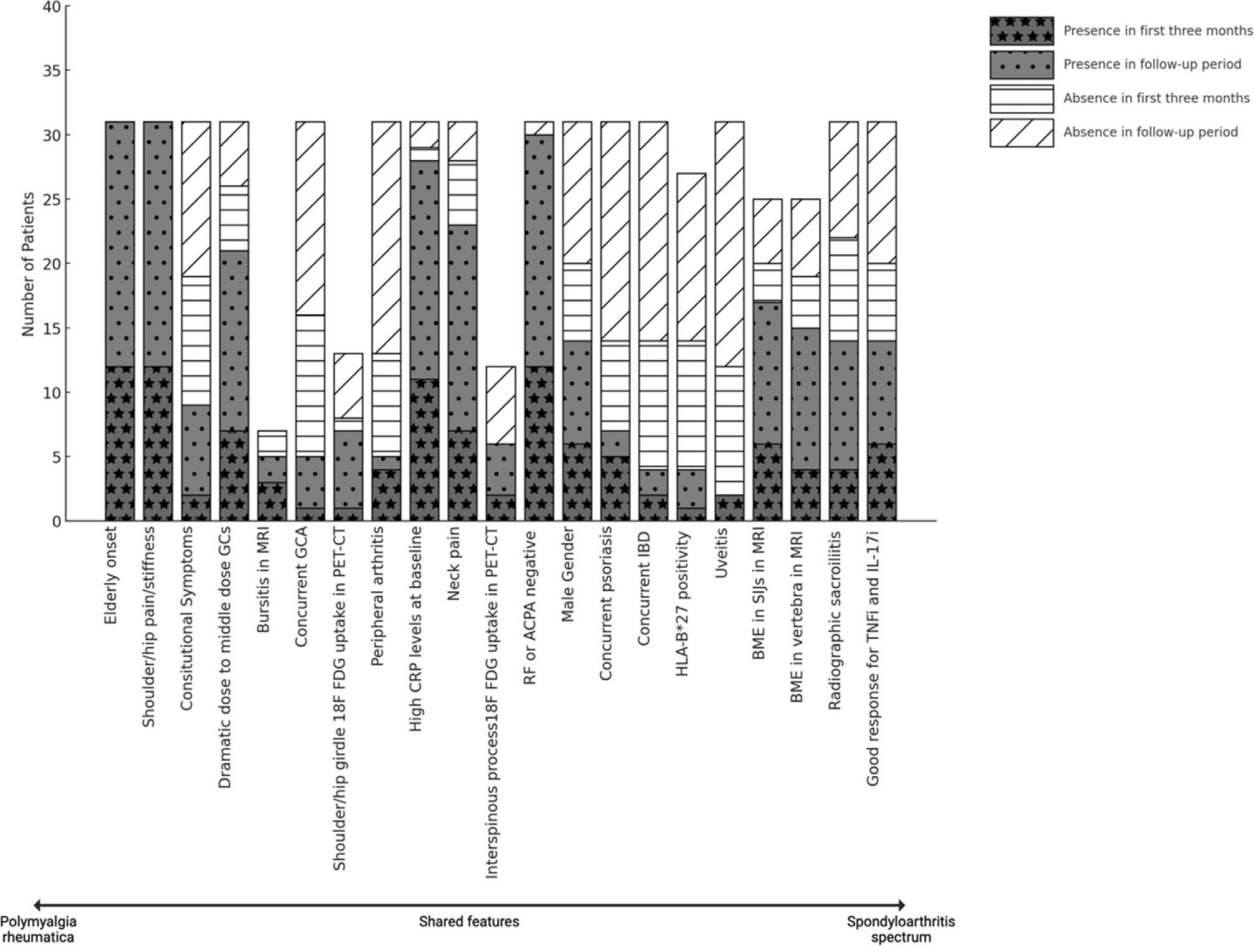
Distributionof symptoms presented by patients according to clinical features in the spectrum of polymyalgia rheumatica and spondyloarthritis. Only patients who underwent the relevant investigations (e.g., MRI, HLA‐B27 testing) were included in each respective column, and positivity rates were calculated based on the number of patients who received that specific test. ACPA, anti–citrullinated protein antibody; BME, bone marrow edema; CRP, C‐reactive protein; FDG, fluorodeoxyglucose; GC, glucocorticoid; GCA, giant cell arteritis; IBD, inflammatory bowel disease; IL‐17i, interleukin‐17 inhibitor; MRI, magnetic resonance imaging; PET‐CT, positron emission tomography/computed tomography; RF, rheumatoid factor; SIJ, sacroiliac joint; TNFi, tumor necrosis factor inhibitor.

**Figure 3:**
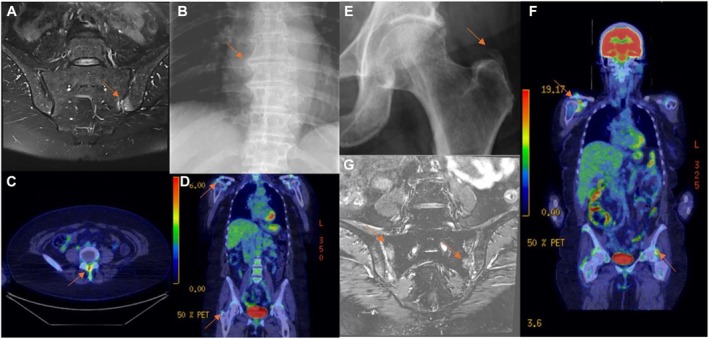
Images of two patients with polymyalgia rheumatica and spondyloarthritic involvement. (A–D) A 54‐year‐old female patient presenting with polymyalgia symptoms. The patient was HLA–B27 negative and had neither psoriasis nor familial history of SpA. Two years following glucocorticoid therapy, all treatments were discontinued without the initiation of any conventional synthetic disease‐modifying antirheumatic drugs. One year after cessation of treatment, the patient presented with recurrent inflammatory back pain, peripheral arthralgias, and elevated C‐reactive protein levels. Upon re‐evaluation, a diagnosis of SpA was confirmed. Subsequently, adalimumab therapy was initiated as part of the management strategy. (A) Bone marrow edema in sacroiliac joints on axial fat‐suppressed T2‐weighted magnetic resonance imaging (red arrow). (B) Unilateral osteophytes in thoracic vertebra (red arrow). (C) Interspinous FDG uptake on axial fused PET/CT (red arrow). (D) Shoulder and hip girdle FDG activity (red arrows) on coronal fused PET/CT, which is the hallmark finding of polymyalgia rheumatica. (E–G) An HLA–B27‐negative 66‐year‐old female patient presenting with polymyalgia symptoms with no SpA history. After glucocorticoid tapering, she had a relapse, and her therapy was changed to golimumab. (E) Trochanteric enthesitis on plain radiography (red arrow). (F) Shoulder and hip girdle involvement, a hallmark finding of polymyalgia rheumatica on coronal fused FDG‐PET/CT (red arrows). (G) Bilateral sacroiliac bone marrow edema on fat‐suppressed T2‐weighted magnetic resonance imaging (red arrows). FDG, fluorodeoxyglucose; PET/CT, positron emission tomography/computed tomography; SpA, spondyloarthritis.

**Figure 4:**
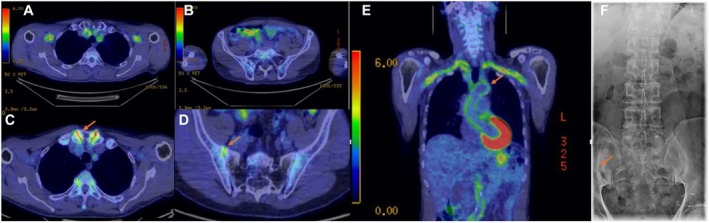
Imagesof a patient with polymyalgia rheumatica, giant cell arteritis, and spondyloarthritis. A 61‐year‐old male patient presented with polymyalgia rheumatica symptoms, chest pain, and constitutional symptoms. PET/CT revealed extensive involvement of the aorta, subclavian, and carotid arteries. High‐dose steroid therapy was initiated and subsequently tapered, and the patient was observed under methotrexate treatment. The patient reported low back pain since the initial presentation. A follow‐up PET/CT scan performed after four years revealed sacroiliitis. (A and B) Initial PET/CT showed no significant sacroiliac or costovertebral joint involvement. (C and D) PET/CT at year four demonstrated sacroiliac, sternoclavicular, and costovertebral joint involvement. (E) Initial PET/CT revealed extensive aortic and subclavian involvement. (F) Sacroiliac radiograph showed bilateral sclerosis (more extensive on the right side) and bilateral stage 2 sacroiliitis based on the modified New York criteria. PET/CT, positron emission tomography/computed tomography. Color figure can be viewed in the online issue, which is available at http://onlinelibrary.wiley.com/doi/10.1002/art.43320/abstract.

Herein, we describe the clinical, imaging, and treatment characteristics of a cohort of patients with SpA spectrum disorders initially presenting as PMR. Some cases were quickly redesignated as SpA based on MRI in particular, but almost half of our cases were only diagnosed as SpA during follow‐up and due to a lack of satisfactory GC response or the inability to taper GCs, which triggered the MRI investigation that supported the SpA diagnosis. As emphasized in recent reviews, an insufficient response to GC therapy in polymyalgia‐like presentations should raise suspicion for alternative diagnoses, including SpA, and may warrant early imaging.^22^ In our study, findings such as increased FDG uptake in the shoulder and hip girdle on PET/CT, tendon sheath involvement on hip MRI, late‐onset presentation, constitutional symptoms, presence of concurrent GCA, and significant improvement with medium‐dose GC treatment extend beyond the typical definitions of “polymyalgic symptoms” or “polymyalgia mimicking.” This clinical picture appears more consistent with an overlap syndrome (Figures 2, 3, 4), which is credible because both conditions show a shared anatomic topography with extra synovial capsular and entheseal involvement. What makes our study especially interesting is that GC‐refractory SpA may respond to anti–interleukin‐17 (anti–IL‐17) therapy, and our findings raise the intriguing possibility that many such cases may in fact have genuine SpA pathologic features on imaging.^23^

Our study collected patient data over 22 years (Supplementary Figure) and is the largest evaluation of the PMR and SpA diagnostic uncertainty. Case reports and small case series have previously hinted at this, including late‐onset SpA presenting with PMR.^24^ Similarly, Elkayam et al and Olivieri et al reported five and seven patients with PMR subsequently diagnosed with SpA, respectively.^12^, ^25^ In the aforementioned study, all patients presented after the age of 50 years, four had radiographic sacroiliitis detected, and six had HLA–B27 positivity.^12^ Specific findings such as sacroiliitis and HLA–B27 positivity are sufficient to classify patients as having axSpA.^17^ However, rather than simply having SpA that mimicked PMR symptomatically, patients in our study actually exhibited many specific features characteristic of PMR, and most were HLA–B27 negative, even when they developed an axSpA phenotype.

For data analysis, we split patients into two groups: one with an early follow‐up recognition of SpA and the other with delayed recognition. Remarkably, both groups exhibited similar clinical characteristics, including isolated enthesitis, HLA–B27 status, clinical and MRI patterns of SpA, and response to therapy (Table 1). Psoriasis and a family history of SpA were more frequently observed in the group diagnosed within the first three months. Although these features are undoubtedly part of the overlap phenotype, their higher prevalence in the early diagnosis group may be attributed to their role in prompting earlier clinical suspicion and imaging for SpA. Further work is needed, and the anatomic overlap of inflamed tissues may be relevant. Indeed, they could alternatively be regarded as subtle clinical cues that enhance the clinician's early awareness and diagnostic precision.

The pathogeneses of PMR and SpA are regarded as quite different, with SpA typically representative of the concept of “MHC‐I‐opathy,” characterized by an inflammatory process driven by major histocompatibility complex (MHC) class I peptide presentation and dominated by IL‐23/17 cytokine pathways.^26^, ^27^ In contrast, PMR is more frequently associated with MHC class II alleles, particularly HLA‐DRB1*04, especially in cases overlapping with GCA; however, this association is more variable in isolated PMR cases, may not be universal across all populations,^28^, ^29^, ^30^ and is characterized by increased IL‐6–producing monocytes and elevated circulating IL‐6 levels.^31^, ^32^ IL‐6–targeted therapies are an important treatment option in PMR,^33^ whereas their use is more restricted and they are still rarely applied in refractory patients with SpA.^34^, ^35^, ^36^ Nevertheless, there are immunologic similarities between PMR and SpA. PMR shows an inclination toward type 17 disease. Neutrophil dysregulation and increased macrophage and monocyte activity, characteristics of psoriatic and SpA disease, are also found in PMR. Neutrophils have been found to be more NETotic than those in healthy controls, which is a strong driver of Th17 polarization. Intriguingly, in contrast to calprotectin levels and the erythrocyte sedimentation rate, levels of neutrophil extracellular traps did not normalize after GC therapy in PMR.^37^ Reductions in Th1 and Treg cell populations relative to Th17 cell populations have been reported, and in patients with GCA compared to controls, Th17 cells with high capacity for IL‐17 production were found to highly infiltrate temporal arterial tissue.^38^ Interestingly, although PMR is not associated with MHC class I, CD8 T cell activation has been identified in patients with active PMR, and it has been postulated that bystander activation of memory CD8 T cells might contribute to PMR pathogenesis.^39^ This and other immunologic similarities may perhaps provide a mechanistic bridge between SpA disease and PMR in predisposed individuals. Indeed, some findings in our patients are more attributable to SpA.

**Figure 5:**
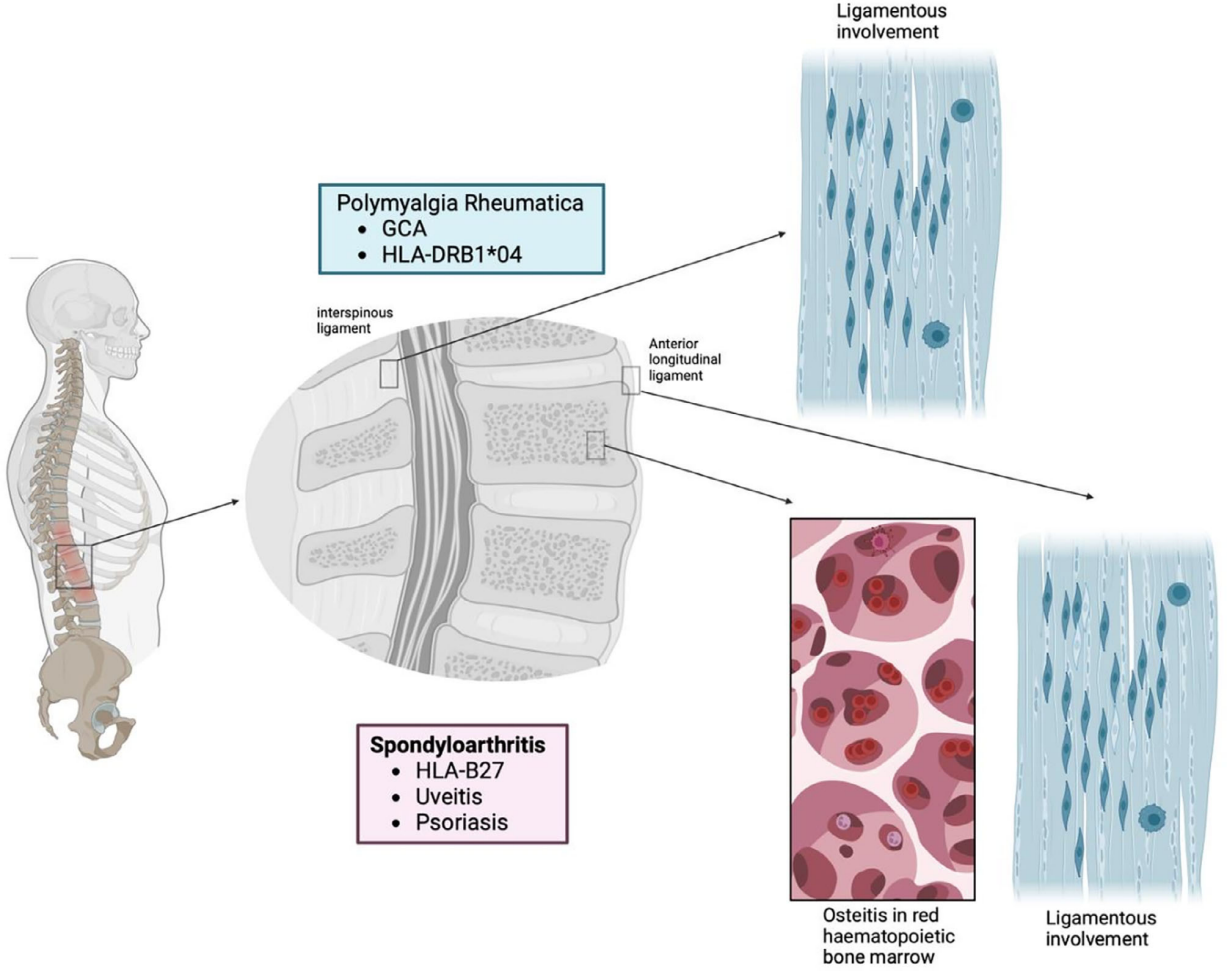
Distinct pathophysiologic patterns and overlap in axial spondyloarthritis, psoriatic arthritis, and polymyalgia rheumatica. In axial spondyloarthritis, osteitis is predominantly observed. However, ligament‐centric involvement represents an alternative pathogenic pathway, particularly in psoriatic arthritis. The shared feature of these two distinct pathogeneses is evolving to new bone formation. Polymyalgia rheumatica, on the other hand, is characterized by soft tissue involvement that does not progress to new bone formation. In our patient cohort, we observed not only soft tissue involvement consistent with polymyalgia rheumatica, as identified clinically and via FDG‐PET/CT, but also bone marrow edema detected on MRI, complementing these clinical findings. Furthermore, the presence of extramusculoskeletal system manifestations in both disease groups unveils a unique overlap syndrome, highlighting the complexity and interplay of these conditions. FDG, fluorodeoxyglucose; GCA, giant cell arteritis; MRI, magnetic resonance imaging; PET/CT, positron emission tomography/computed tomography. Color figure can be viewed in the online issue, which is available at http://onlinelibrary.wiley.com/doi/10.1002/art.43320/abstract.

Though HLA–B27 positivity was infrequent, osteitis was detected at a notably high rate. Osteitis in SIJs is an unusual finding for PMR^5^, ^7^ but is a distinctive finding in SpA,^15^ particularly the BME in the SIJs detected by MRI (Figure 5).^40^ The BME observed on MRI in our patients meets the ASAS criteria for sacroiliitis, and radiographic sacroiliitis was present in almost half of the patients.^19^, ^20^ Additionally, the presence of SpA spectrum disorders, such as uveitis, psoriasis, IBD, a family history of SpA, peripheral enthesitis, and peripheral arthritis (especially in the lower extremities), is a well‐accepted finding in the diagnosis of SpA.^17^ Against these clear differences are the reports of anatomic territory convergence, including the proclivity for interspinous ligament soft tissue inflammation in both SpA (including PsA)^41^ and PMR, which may be relevant for the overlapping features that we report (see graphical abstract).

In our study, increased shoulder and hip girdles, FDG uptake, tendon sheath involvement on hip MRI, late‐onset presentation, constitutional symptoms, presence of concurrent GCA, and significant improvement with medium‐dose GC treatment extend beyond the usual concepts of “polymyalgic symptoms” or “polymyalgia mimics.” This clinical picture appears more consistent with an overlap syndrome of SpA and PMR in some cases (Figures 2 and 3). Shared features across age groups, including capsular/entheseal joint involvement and vascular involvement of aorta and branches in settings such as PMR plus GCA and in SpA, do raise the possibility of an occasional genuine overlap rather than two distinct diseases (Figure 4). The optimal treatment strategy for this overlap syndrome remains unclear, but it is quite possible the steroid‐refractory PMR that responds to anti–IL‐17 therapy could represent a novel PMR subgroup that manifests as SpA.^23^ As our understanding of disease pathogenesis continues to accrue, the taxonomy of disease may need to be updated to stratify patients to the optimal treatment strategy.^42^ The concept of an overlap between SpA and PMR mirrors recent discourse in relation to difficulty in defining a clear dividing line between PMR and late‐onset RA^13^ or between PMR and GCA.^43^ The promising recent advancements in the efficacy of IL‐17 blockade in the treatment of GCA may hint at pathogenic overlaps that extend beyond mere topographic similarities.^23^

One‐third of our patients were started on bDMARDs during follow‐up, including anti‐TNFα and anti–IL‐17, which are both first‐line options for axSpA treatment. MTX is not advocated in the ASAS‐EULAR recommendations for the management of axSpA,^44^ but five patients in our cohort continued successful MTX monotherapy. Additionally, 11 patients were no longer taking immunosuppressants at their last follow‐up, indicating that this pattern of disease will eventually remit. Nevertheless, this PMR–SpA overlap is characterized by prolonged GC therapy use, and early identification could certainly facilitate prompt DMARD or bDMARD use to minimize steroid side effects.

In conclusion, we report PMR presentation of late‐onset SpA subsequently confirmed by MRI‐detected BME associated with the inability to taper GCs. In PMR cases with refractory symptoms or difficult GC reduction, these findings raise the possibility that MRI may have a role in more accurate identification of a subset of patients with PMR–SpA overlap, with potential treatment implications. Given the heterogeneity in disease trajectories and diagnostic delay in this cohort, future prospective observational studies are warranted to better characterize the frequency, natural history, and optimal management strategies of this PMR–SpA overlap phenotype. Looking to the future, we suggest that considering PMR diagnosis only as a problem of “mimics/misdiagnosis” may no longer serve us well; we propose that more precise subphenotyping of patients initially diagnosed with PMR may identify opportunities for moving beyond the era of GC monotherapy for this underserved patient group.

## AUTHOR CONTRIBUTIONS

All authors contributed to at least one of the following manuscript preparation roles: conceptualization AND/OR methodology, software, investigation, formal analysis, data curation, visualization, and validation AND drafting or reviewing/editing the final draft. As corresponding author, Dr McGonagle confirms that all authors have provided the final approval of the version to be published and takes responsibility for the affirmations regarding article submission (eg, not under consideration by another journal), the integrity of the data presented, and the statements regarding compliance with institutional review board/Declaration of Helsinki requirements.

## Supporting information


**Disclosure form**.

**Supplementary Figure 1:** Annual Diagnosis Counts of PMR & SpA Patients.
