# Supplementary material for: Late‐Onset Spondyloarthritis Presenting as Glucocorticoid‐Resistant Polymyalgia Rheumatica: A Hitherto Underappreciated Entity in Which Tumor Necrosis Factor or Interleukin ‐17 Blockade May Have a Therapeutic Role
Source: Arthritis Rheumatol. 2025 Dec 8;78(1):131–40. doi: 10.1002/art.43320 (PMC12854004; doi:10.1002/art.43320)
Supplement: Supplementary file 2 — Supplementary Figure 1: Annual Diagnosis Counts of PMR & SpA Patients. [file ART-78-131-s001.docx]

**Supplementary Figure.** Annual Diagnosis Counts of PMR & SpA Patients
